# Elevated eosinophil level predicted long time to next treatment in relapsed or refractory myeloma patients treated with lenalidomide

**DOI:** 10.1002/cam4.2828

**Published:** 2020-01-16

**Authors:** Kazuhito Suzuki, Kaichi Nishiwaki, Tadahiro Gunji, Mitsuji Katori, Hidekazu Masuoka, Shingo Yano

**Affiliations:** ^1^ Division of Clinical Oncology and Hematology Department of Internal Medicine The Jikei University School of Medicine Tokyo Japan; ^2^ Division of Clinical Oncology and Hematology Department of Internal Medicine The Jikei University Kashiwa Hospital Kashiwa Chiba Japan

**Keywords:** chemotherapy, hematological cancer, immunology

## Abstract

Lenalidomide is an immunomodulatory drug that is administered commonly in patients with relapsed or refractory multiple myeloma (RRMM). Eosinophils have immunological functions, for instance, in allergic diseases and asthma. The purpose of this study was to investigate the clinical significance of elevated eosinophil levels in patients with RRMM treated with lenalidomide. A total of 59 patients were included. Elevated eosinophil level was defined as an increase in the eosinophil count of ≥250/µL from the eosinophil count on day 1 during the first cycle. The percentage of patients with elevated eosinophil levels was 22.0%. The overall response ratio in the elevated eosinophil group and nonelevated eosinophil group was 84.6% and 63.0% (*P* = .189), respectively. The median time to next treatment (TTNT) in the elevated eosinophil group was significantly longer than that in the nonelevated group (40.3 months vs 8.4 months; *P* = .017). Additionally, TTNT in the elevated eosinophil group with partial response (PR) or better was significantly longer than that in the nonelevated eosinophil group with PR or better (40.3 months vs 11.9 months; *P* = .021). We concluded that elevated eosinophil levels were frequently observed and might predict a longer TTNT in patients with RRMM treated with lenalidomide.

## Graphical abstract

Elevated eosinophils predicted significantly long time to next treatment in relapsed or refractory multiple myeloma patients treated with lenalidomide. Elevated eosinophils might show an immunological effect of lenalidomide. We considered that NKG2D and IL‐2 were associated with elevated eosinophils according to the literature review.

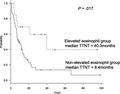

## 1 INTRODUCTION

Multiple myeloma comprises a heterogeneous group of plasma cell neoplasms, which vary in terms of their morphology, phenotype, molecular biology, and clinical behavior. Although the development of novel agents, such as bortezomib, thalidomide, and lenalidomide, has improved the prognosis of patients with this condition over the last decade, multiple myeloma remains incurable.^1^ Studies on multiple myeloma have identified a large number of prognostic factors for survival, which include the disease stage according to the International Staging System (ISS) 2 or the Durie‐Salmon staging system 3 and the detection of high‐risk cytogenetic abnormalities using fluorescence in situ hybridization 4, 5, 6, 7, 8 in newly diagnosed multiple myeloma. Lenalidomide is an immunomodulatory drug (IMiD) that directly targets myelomas and stimulates an immunological response.^9^, ^10^ It is administered in combination with antimyeloma agents, such as carfilzomib,^11^ ixazomib,^12^ elotuzumab,^13^ daratumumab,^14^ bortezomib,^15^ and corticosteroids,^16^, ^17^ in patients with relapsed or refractory multiple myeloma (RRMM). However, predictive factors, which are used in clinical practice, for survival were not detected in patients with RRMM treated with lenalidomide. Cereblon (CRBN) level was difficult to analyze in clinical practice, although CRBN was identified as a prognostic factor for clinical outcome of lenalidomide in patients with RRMM.^18^, ^19^

Eosinophils are types of blood cells that work immunologically, for instance, in allergic diseases and asthma.^20^, ^21^, ^22^ Clinical features of eosinophilia in patients with multiple myeloma treated with lenalidomide have not yet been analyzed. Therefore, this retrospective study aimed to investigate the clinical significance of elevated eosinophil levels in patients with RRMM treated with lenalidomide‐containing regimens.

## 2 MATERIALS AND METHODS

We reviewed medical records of patients with RRMM treated with a lenalidomide‐containing regimen at the Jikei University Kashiwa Hospital between November 2010 and June 2018, and these patients were followed up until March 2019. This study was approved by the independent ethics committee/institutional review board of our institution.

### 2.1 Patients

Patients were included if they were older than 20 years and had RRMM for which they had previously undergone one or more regimens of chemotherapy. Relapse and refractory disease was defined according to the International Myeloma Working Group criteria.^23^ Patients treated continuously with lenalidomide, such as maintenance treatment after autologous stem cell transplantation and initial treatment, were excluded from the analysis.

### 2.2 Treatment and response assessment

A total of 59 patients received standard salvage therapy regimens, which included lenalidomide plus dexamethasone (LD); bortezomib, lenalidomide, and dexamethasone (BLD); melphalan, lenalidomide, and prednisolone; elotuzumab, lenalidomide, and dexamethasone (ELD); ixazomib, lenalidomide, and dexamethasone (ILD); or daratumumab, lenalidomide, and dexamethasone (DLD). Disease response was assessed according to the International Myeloma Working Group criteria.^23^

### 2.3 Prognostic factors

Elevated eosinophil level was defined as an increase in the eosinophil count of ≥250/µL from the eosinophil count on day 1 during the first cycle. The following parameters were recorded and evaluated in each group: age, sex, subtype of monoclonal protein, interval from diagnosis, number of prior chemotherapy regimens, prior autologous stem cell transplantation, triplet regimen including lenalidomide, dose of lenalidomide, estimated glomerular filtration rate, serum C‐reactive protein level, serum lactate dehydrogenase level, and treatment response. The presence of cytogenetic abnormalities and clinical stage by ISS was not analyzed because these data were not evaluated in the majority of patients when they started undergoing lenalidomide‐containing salvage treatment.

### 2.4 Statistical analysis

The primary endpoint was to evaluate the association between elevated eosinophil levels and time to next treatment (TTNT). TTNT was calculated from the initiation of salvage treatment to the start date of the next treatment. Fisher's exact test was used to compare various parameters between the elevated and nonelevated eosinophil groups. The mean initial dose of lenalidomide between the elevated and nonelevated eosinophil groups was analyzed using a *t* test. Actuarial survival analysis was performed using the Kaplan‐Meier method, and the resultant curves were compared using the log‐rank test. All prognostic variables were considered by multivariate analysis for survival. The latter was performed using Cox regression analysis. Finally, TTNT was evaluated in three groups: PR or better with elevated eosinophil, PR or better without elevated eosinophil, and stable or progressive disease (PD). All reported *P*‐values are two‐sided, and *P*‐values <.05 were considered to be statistically significant. All statistical analyses were performed using EZR (Saitama Medical Center, Jichi Medical University), which is a graphical user interface for r (the R Foundation for Statistical Computing).^24^ More precisely, it is a modified version of R Commander that incorporates frequently used biostatistical functions.

## 3 RESULTS

### 3.1 Patients and elevated eosinophil levels

Fifty‐nine patients were included in this study. Patient characteristics are shown in Table 1. The median age of patients was 73 years (range, 45‐89 years). The median interval between diagnosis and starting lenalidomide‐containing salvage treatment was 25.9 months (range, 1.7‐90.4 months). The number of patients who experienced relapsed and refractory disease was 53 (90%) and 6 (10%), respectively. The number of patients who received lenalidomide‐containing salvage treatment as a second‐line treatment was 28 (47%). Regarding salvage chemotherapy, 39 (66%), 8 (14%), 4 (7%), 3 (5%), 3 (5%), and 2 (3%) patients received LD, ELD, DLD, ILD, BLD, and MLD treatment, respectively.

**Table 1:** Patient characteristics

	All (n = 59)	Elevated eosinophil group (n = 13)	Nonelevated eosinophil group (n = 46)	*P*‐value
Age (y)				
≥70	33	6	27	.531
≤69	26	7	19	
Sex				
Male	30	7	23	.999
Female	29	6	23	
Subtype of M protein				
IgG type	32	6	26	.544
Non‐IgG type	27	7	20	
IgA	13	2	11	
BJP	10	4	6	
IgD	4	1	3	
ISS stage				
1 or 2	33	6	27	.728
3	20	5	15	
Unknown	6	2	4	
Disease status				
Relapsed	53	13	40	.322
Refractory	6	0	6	
Number of prior chemotherapies				
1	28	9	19	.116
≥2	31	4	27	
Prior bortezomib				
Yes	49	12	37	.432
No	10	1	9	
Prior thalidomide				
Yes	16	1	15	.090
No	43	12	31	
Prior autologous stem cell transplantation				
Yes	9	1	8	.668
No	50	12	38	
Interval from diagnosis (y)				
≥2	30	5	25	.360
<2	29	8	21	
Lenalidomide‐containing regimen				
Triplet	20	6	14	.332
Doublet	39	7	32	
Initial dose of lenalidomide				
≥15 mg/body	24	5	19	.999
<15 mg/body	35	8	27	
eGFR (mL/min)				
≥40	43	8	35	.311
<40	16	5	11	
Serum LDH level				
≥UNL	16	3	13	.999
<UNL	46	10	36	
Skin rash as adverse event				
Yes	13	2	11	.713
No	46	11	35	

Abbreviations: BJP, Bence Jones protein; eGFR, estimated glomerular filtration rate; ISS, International Staging System; LDH, lactate dehydrogenase; UNL, upper normal limit.

**Figure 1:**
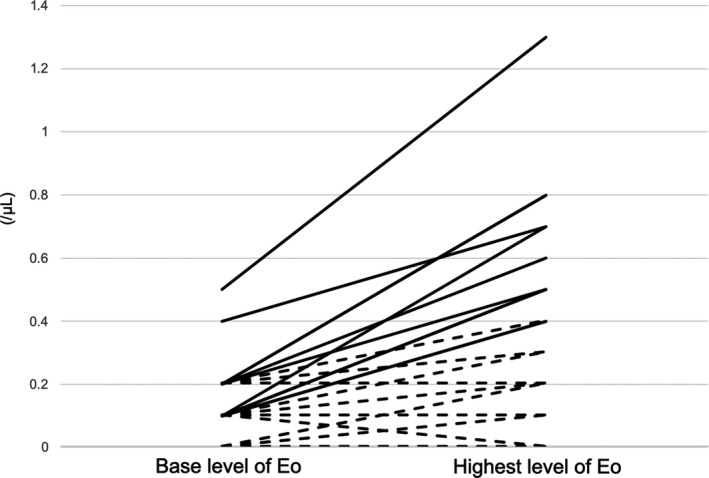
The kinetics of change in eosinophil count. An increase in the eosinophil count of >250/μL is observed in 13 patients (dotted solid lines), that of <250/μL is observed in 22 patients, and no change in the basal eosinophil level is observed in 23 patients; a decrease in the eosinophil count from the baseline is observed in only one patient (dotted line)

The kinetics of the change in eosinophil count in each patient is shown in Figure 1. There were similar patterns of eosinophil variation between patients, and decreased eosinophil levels were observed in only one patient. The number of patients in the elevated eosinophil and nonelevated eosinophil groups was 13 (22%) and 46 (78%), respectively. The median day of elevated eosinophil was 15 (range, 8‐28). There were no significant differences in patient characteristics between the elevated eosinophil and nonelevated eosinophil groups (Table 1). The mean initial doses of lenalidomide between the elevated eosinophil and nonelevated eosinophil groups were 14.6 and 13.5 mg/body, respectively (*P* = .621). The incidence of skin rash was not significantly different between the elevated and nonelevated eosinophil groups (15.4% vs 23.9, *P* = .713).

### 3.2 Response and survival

The overall response ratio was 67.8% in all patients. Fourteen patients achieved very good partial response or better; 26 patients achieved partial response (PR); 12 patients exhibited stable disease; and seven patients had PD. Patients’ responses to lenalidomide‐containing salvage treatment are shown in Table 2. The overall response ratios in the elevated and nonelevated eosinophil groups were 84.6% and 63.0%, respectively (*P* = .189). There were no patients with PD in the elevated eosinophil group, although this was not a statistically significant observation (*P* = .330).

**Table 2:** Response between the elevated eosinophil and nonelevated eosinophil groups

	All (n = 59)	Elevated eosinophil group (n = 13)	Nonelevated eosinophil group (n = 46)	*P*‐value
VGPR or better	14	5	9	
PR	26	6	20	
SD	12	2	10	
PD	7	0	7	
VGPRR	23.7%	38.5%	19.6%	.266
ORR	67.8%	84.6%	63.0%	.189
CBR	88.1%	100%	84.8%	.330

Abbreviations: CBR, clinical benefit rate; ORR, overall response rate; PD, progressive disease; PR, partial response; SD, stable disease; VGPR, very good partial response; VGPRR, very good partial response rate.

**Figure 2:**
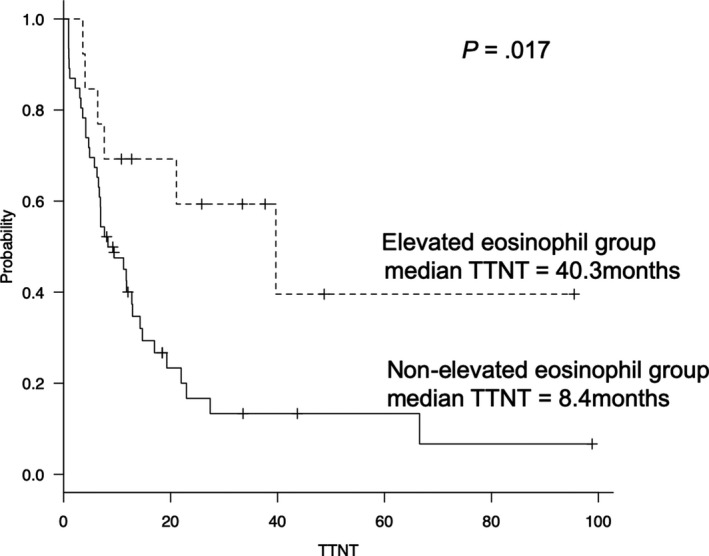
The median TTNT in the elevated eosinophils and nonelevated eosinophils groups. In the forty‐seven patients treated with lenalidomide‐containing regimen, the median TTNT in the elevated eosinophil group was longer than those in the nonelevated eosinophil group, significantly (40.3 and 7.0 months, *P* = .034)

**Figure 3:**
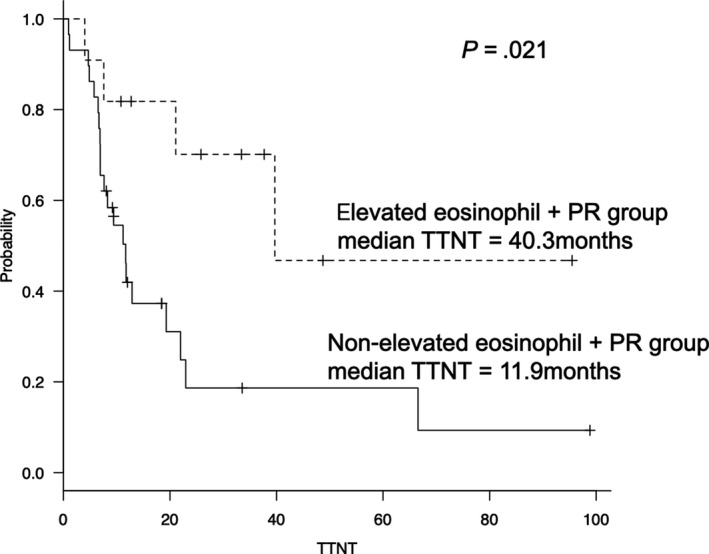
The median TTNT in the elevated eosinophils with PR, nonelevated eosinophils with PR, and without PR groups. In the patients treated with lenalidomide‐containing regimen, the median TTNT in the elevated eosinophil group with PR was longer than those in the nonelevated eosinophil group with PR and the patients with PR, significantly (40.3 vs 9.6 and 4.2 months, *P* = .003)

**Figure 4:**
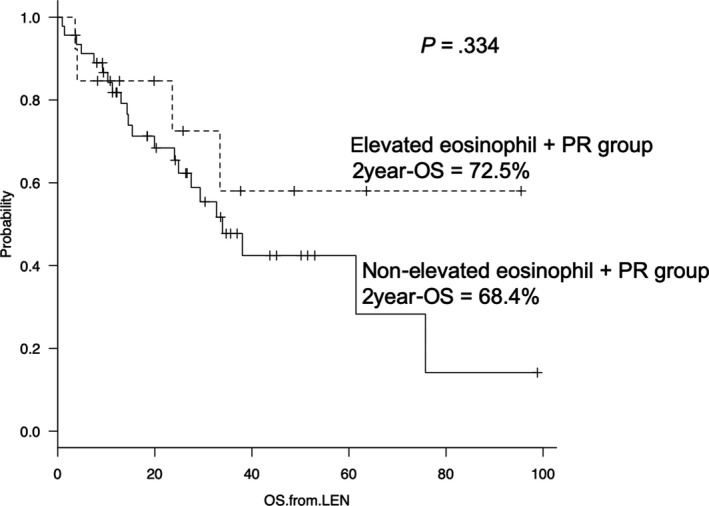
The median OS in the elevated eosinophils with PR, nonelevated eosinophils with PR, and without PR groups. In the patients treated with lenalidomide‐containing regimen, the median OS in the elevated eosinophil group with PR tended to be longer than those in the nonelevated eosinophil group with PR and the patients with PR, significantly (not reached vs 38.6 and 25.2 months, *P* = .063)

The median follow‐up period for survival was 24.4 months. The median TTNT in the elevated eosinophil group was significantly longer than that in the nonelevated eosinophil group (40.3 and 8.4 months, respectively; hazard ratio, 0.362; 95% confidence interval [CI], 0.154‐0.867; *P* = .017; Figure 2). In the 39 patients treated with LD, the median TTNT in the elevated eosinophil group was longer than that in the nonelevated eosinophil group (40.3 and 8.3 months, respectively; *P* = .078). The other significant prognostic factors for shorter TTNT were refractory disease and prior three or more treatments (*P* = .007 and 0.029). Significant association between TTNT and the other patient characteristics was not observed. A summary of univariate analysis for the TTNT is shown in Table 3. In multivariate analysis, significant predictors for longer TTNT were elevated eosinophil levels (hazard ratio, 0.401; 95% CI, 0.166‐0.969; *P* = .042) and refractory disease (hazard ratio, 2.575; 95% CI, 1.055‐6.286; *P* = .038). A summary of multivariate analysis for the TTNT is shown in Table 4. Finally, the median TTNT in the elevated eosinophil group with PR or better was significantly longer than that in the nonelevated eosinophil group with PR or better (40.3 vs 11.9 months, *P* = .021; Figure 3). Additionally, the 2‐year overall survival (OS) rate was similar between the elevated eosinophil and nonelevated eosinophil groups (72.5% vs 68.4%; *P* = .334; Figure 4).

**Table 3:** Univariate analysis of time to next treatment

	Number of patients	Median TTNT	95% CI	*P*‐value
Elevated eosinophils				
Yes	13	40.3	6.4‐NA	.0174
No	46	8.4	6.3‐13.1	
Age (y)				
≥70	33	11.9	6.3‐17.2	.743
<70	26	12.2	6.5‐23.3	
Sex				
Male	30	12.9	6.6‐21.4	.644
Female	29	11.4	6.3‐17.2	
ISS stage				
1 or 2	33	11.4	4.7‐14.9	.322
3	20	9.8	6.3‐40.3	
Refractory disease				
Yes	6	4.2	0.9‐NA	.007
No	53	11.9	7.0‐27.8	
Interval from diagnosis (y)				
≥2	29	11.9	7.0‐17.2	.742
<2	30	8.4	4.7‐23.3	
Number of prior chemotherapies				
1	28	23.3	7.0‐67.5	.0292
≥2	31	7.7	4.9‐13.1	
Lenalidomide‐containing regimen				
Triplet	20	11.9	5.8‐NA	.672
Doublet	39	11.4	6.3‐19.6	
Dose of lenalidomide (mg)				
≥15	24	11.9	6.8‐23.3	.598
<15	35	9.5	6.3‐19.6	
Skin rash as adverse event				
Yes	12	11.9	2.2‐NA	.346
No	45	11.4	6.6‐17.2	

Abbreviations: CI, confidence interval; eGFR, estimated glomerular filtration rate; ISS, International Staging System; LDH, lactate dehydrogenase; UNL, upper normal limit.

**Table 4:** Multivariate analysis of time to next treatment

	Hazard ratio	95% CI	*P*‐value
Elevated eosinophils			
No	1		
Yes	0.401	0.166‐0.969	.042
Refractory disease			
No	1		
Yes	2.575	1.055‐6.286	.038
Number of prior chemotherapies			
1	1		
≥2	1.650	0.875‐3.131	.123

Abbreviation: CI, confidence interval.

## 4 DISCUSSION

The clinical significance of elevated eosinophil levels in patients with RRMM receiving lenalidomide‐containing treatment has not been analyzed until now. The percentage of patients with elevated eosinophil levels was 22.0%. Elevated eosinophil levels were not associated with higher response rate but were significantly associated with longer TTNT. Additionally, elevated eosinophil levels predicted longer TTNT in patients with PR. Thus, we hypothesized that elevated eosinophil levels might be associated with immunological activity in patients treated with lenalidomide.

In the MM‐009 and MM‐010 trials, the time to progression (TTP) and OS in patients treated with LD were significantly longer than those in patients treated with high‐dose dexamethasone.^16^, ^17^ In subgroup analysis, treatment with a previous regimen, low beta‐2 microglobulin levels, and low bone marrow plasmacytosis predicted longer TTP in patients treated with LD. Prior thalidomide treatment did not affect outcome in patients treated with LD. However, in these trials, eosinophil levels were not studied.

Lenalidomide is an antimyeloma agent with direct antitumor and immunomodulating activities. CRBN is the most important molecular target of the direct antimyeloma activity of lenalidomide. Lenalidomide binds to CRBN and activates the enzymatic activity of the CRBN E3 ubiquitin ligase complex. Ikaros (IKZF1) and Aiolos (IKZF3), which are important B‐cell transcriptional factors, are modified with ubiquitin molecules and degraded by the proteasome. Degradation of IKZF1 and IKZF3 causes the downregulation of interferon regulatory factor 4 and cMYC, which play a role in proliferation and survival of myeloma cells.^25^, ^26^, ^27^ On the contrary, the immunological activity of lenalidomide comprises co‐stimulation of T cells,^28^, ^29^ enhancement of the type 1 T helper–mediated immune response,^9^, ^30^, ^31^ enhancement of natural killer (NK) and NK T cells,^10^, ^32^, ^33^, ^34^, ^35^ and inhibition of regulatory T cells.^36^

Eosinophils play a role in type 2 T helper–type immune responses.^37^ Interleukin (IL)‐3 and granulocyte‐macrophage colony‐stimulating factor (GM‐CSF) stimulate the proliferation of neutrophils, basophils, and eosinophils in a nonspecific manner, although IL‐5 specifically stimulates eosinophil production.^38^ Eosinophils are recruited to inflammatory sites and produce several cytokines, such as IL‐4,^39^ vascular endothelial cell growth factor,^40^ transforming growth factor‐α, and TGF‐β1.^41^, ^42^ Partial activation of eosinophils is promoted by cytokines and growth factors and facilitates tissue repair and immune regulation. In contrast, full activation of eosinophils by inflammatory mediators can cause inflammation and tissue damage.^37^ In patients undergoing allogeneic hematopoietic stem cell transplantation for hematological disorders, eosinophilia was associated with a high incidence of chronic graft‐versus‐host disease and long OS.^43^ In our study, elevated eosinophils predicted longer TTNT in patients with PR or better. Thus, elevated eosinophils might be recognized via immunological activity and associated with good clinical outcome.

The association between the immunological activity of lenalidomide and elevated eosinophil levels has not been studied. We considered that the NK group 2D (NKG2D) 44, 45, 46, 47 and IL‐2 48, 49 were associated with proliferation of eosinophils in patients treated with lenalidomide. Shafi et al and Strid et al reported that NKG2D upregulation increased eosinophil levels in mice, confirming an association between NKG2D ligands, the innate lymphoid stress surveillance response, and atopy.^44^, ^45^ The activation of NK cells depends significantly on NK receptor member D of the lectin‐like receptor family, such as NKG2D.^46^, ^47^ IMiDs enhanced the expression of NKG2D on NK cells in patients with myeloma treated with lenalidomide and pomalidomide in 7‐14 days.^50^, ^51^ NKG2D recognizes NKG2D ligands on tumor cells, and tumors downregulate NKG2D ligand expression to prevent immune effect.^52^, ^53^ Thus, NKG2D plays an important role in antibody‐dependent cellular cytotoxicity (ADCC), and IMiDs activate ADCC via the upregulation of NKG2D expression on NK cells. Additionally, in another cohort at our single‐center experience, the incidence rates of elevated eosinophils in patients treated with elotuzumab (n = 21) and daratumumab (n = 22) plus Ld were 38.1% and 4.5%, respectively (data were not shown). Pazina et al^54^ demonstrated that elotuzumab increased NKG2D level in SKOV cells, which was a human ovarian cancer cell line, and expressed SLAM family member 7. Elotuzumab enhanced NK cytotoxicity to myeloma cells in an independent CD16 expression manner when the expression level of NKG2D was high on NK cells.^54^ On the contrary, daratumumab decreases the number of NK cells in patients with RRMM.^55^ Daratumumab decreased CD38^+^ NK cells significantly, which are present in the majority of the population, accounting for approximately 85%, although daratumumab did not decrease CD38^‐/low^ NK cells.^56^ However, CD38^‐/low^ NK cells play an important role in ADCC compared with CD38^+^ NK cells. Thus, daratumumab acts on ADCC by CD38^‐/low^ NK cells. On the contrary, the expression of NKG2D was not significantly different between CD38^+^ and CD38^‐/low^. Therefore, ADCC works well, although NKG2D expression was low in patients treated with daratumumab. This evidence might support our hypothesis regarding the association between elevated eosinophil levels and ADCC in patients treated with lenalidomide. IL‐2 is a cytokine that is associated with the activation of several immune cells, including T, B, and NK cells.^48^ The association between eosinophilia and IL‐2 levels might suggest that eosinophilia is pathogenically associated with T‐cell activation. In vitro, recombinant IL‐2 increased eosinophil levels in human myeloma cells.^49^ Additionally, IL‐2 therapy in patients with melanoma increases eosinophil levels.^57^ In one study, the beneficial effect of thalidomide was demonstrated to be dependent on IL‐2 induction of natural cytotoxicity.^58^ Lenalidomide releases IL‐2 from T cells via the activation of the CRBN‐CRL4 E3 ubiquitin ligase to degrade the IKZF1 and IKZF3.^59^ IL‐2 has an important role in the immunological activity of lenalidomide and is associated with elevated eosinophil levels. Therefore, we considered that elevated eosinophil levels might predict prolonged TTNT in patients treated with lenalidomide.

In our study, the incidence of any grade of skin rash was 20.3%, and there was no significant correlation between the skin rash and elevated eosinophils. In MM‐009 and 010 trials, the incidence of any grade of skin rash was 16.9% and 9.7%, respectively. In FIRST trial, the incidence of any grade of skin rash was 12.8% in patients with newly diagnosed myeloma (NDMM) who underwent treatment with LD.^60^ In contrast, in MM‐025 trial, a phase 2 trial for LD in Japanese patients with NDMM, the incidence of any grade of skin rash was 61.5%, and the incidence of grades 3 to 4 of skin rash was 15.4%.^61^ Kojima et al^62^ reported that skin rash was a predictor for long‐term progression free survival and OS in patients with MM who underwent treatment with lenalidomide; in their retrospective study, the incidence of any grade skin rash was 30.2%, which suggests that the incidence of skin rash may be higher in Japanese patients with myeloma who undergo treatment with lenalidomide. Nevertheless, those authors did not report an association between the skin rash and elevated eosinophils. In our study, the elevated and nonelevated eosinophil groups showed similar incidence of skin rash (15.4% vs 23.9%; *P* = .713), and the skin rash group and nonskin rash group showed similar TTNT (11.4 vs 11.9 months; *P* = .713). Grades 1, 2, and 3 of skin rash showed incidence of 13.6% (n = 8), 5.1% (n = 3), and 1.7% (n = 1), respectively, and two cases of grade 2 and one case of grade 3 skin rash belonged to the nonelevated eosinophil group. Based on these results, we considered that skin rash was associated with both lenalidomide and other drugs, such as trimethoprim‐sulfamethoxazole used as prophylaxis for pneumocystis.

There were several limitations to this study. First, there were variations in patient characteristics, including the lenalidomide‐containing regimens and the number of prior chemotherapy regimens they had received. To improve the analysis, studying the association between elevated eosinophil levels and clinical outcomes in newly diagnosed patients treated with LD is significantly required. Second, we did not evaluate other molecules that contribute to eosinophilia, such as IL‐2, IL‐3, IL‐5, GM‐CSF, and NKG2D. These molecules play an important role in immune response in patients treated with lenalidomide, as described. Finally, this is a small retrospective study. To improve our understanding, the association between elevated eosinophils and clinical outcome should be analyzed in large‐scale prospective trial.

In conclusion, elevated eosinophil levels were not frequently observed in patients, with 22.0% demonstrating elevated eosinophil levels. TTNT was significantly longer in the elevated eosinophil group than that in the nonelevated eosinophil group, although the response rate was similar between the two groups. In patients with PR or better, TTNT in the elevated eosinophil group was significantly longer than that in the nonelevated eosinophil group. Thus, elevated eosinophil levels might predict an improved immune response to lenalidomide. However, as our sample size is small, larger‐scale studies are required to increase our understanding of how to best treat these patients.
